# *Escherichia coli* Shiga Toxin Mechanisms of Action in Renal Disease

**DOI:** 10.3390/toxins2122769

**Published:** 2010-12-02

**Authors:** Tom G. Obrig

**Affiliations:** Department of Microbiology and Immunology, School of Medicine, University of Maryland, 685 W. Baltimore St., HSF I Suite 380, Baltimore, MD 21201, USA; Email: tobrig@som.umaryland.edu; Tel.: +1-410-706-6917; Fax: +1-410-706-2025.

**Keywords:** Shiga toxin, kidney, HUS, Gb3, animal model, renal failure, *E. coli*

## Abstract

Shiga toxin-producing *Escherichia coli* is a contaminant of food and water that in humans causes a diarrheal prodrome followed by more severe disease of the kidneys and an array of symptoms of the central nervous system. The systemic disease is a complex referred to as diarrhea-associated hemolytic uremic syndrome (D^+^HUS). D^+^HUS is characterized by thrombocytopenia, microangiopathic hemolytic anemia, and acute renal failure. This review focuses on the renal aspects of D^+^HUS. Current knowledge of this renal disease is derived from a combination of human samples, animal models of D^+^HUS, and interaction of Shiga toxin with isolated renal cell types. Shiga toxin is a multi-subunit protein complex that binds to a glycosphingolipid receptor, Gb3, on select eukaryotic cell types. Location of Gb3 in the kidney is predictive of the sites of action of Shiga toxin. However, the toxin is cytotoxic to some, but not all cell types that express Gb3. It also can cause apoptosis or generate an inflammatory response in some cells. Together, this myriad of results is responsible for D^+^HUS disease.

## Abbreviations

TMA: Thrombotic microangiopathic anemia
D^+^HUS: Diarrhea-associated hemolytic uremic syndrome (typical HUS)
D^-^HUS: Non-diarrhea hemolytic uremic syndrome (atypical HUS)
TTP: Thrombotic thrombocytopenic purpura
E. coli: *Escherichia coli*
STEC: Shiga toxin-producing E. coli
EHEC: Enterohemorrhagic E. coli
VTEC: Verotoxin-producing E. coli
E. coli O157:H7: The most common type of STEC as a cause of D^+^HUS
Stx: Shiga toxin (a generic term in this paper), same as Verotoxin, previously Shiga-like toxin.
Stx1: Shiga toxin type 1
Stx2: Shiga toxin type 2, most common among clinical STEC isolates
VT: Verotoxin, same as Shiga toxin
HUVEC: human umbilical vein endothelial cells
PTC: proximal tubule cells
IHC: immunohistochemistry
VEGF: Vascular endothelial growth factor
TNF: Tumor necrosis factor
vWf: von Willebrand factor
MCP-1: Monocyte chemotactic protein type 1
MIP-2: Monocyte inflammatory protein

## 1. Introduction

This review focuses on the kidney disease caused by Shiga toxin-producing E. coli, referred to as diarrhea-associated hemolytic uremic syndrome (D^+^HUS). An attempt is made to relate the clinical course of D^+^HUS in humans with the experimental studies *in vitro*, and *in vivo*, *i.e.*, with animal models of D^+^HUS. Brief descriptions are provided for the structure and function of the Shiga toxins (Stx1, Stx2) and its receptor (Gb3). More emphasis has been placed on the interaction of Stx1 and Stx2 with isolated renal cells and on kidney disease that develops in the animal models. In addition, the role of *E.* coli LPS in D^+^HUS is addressed. This review does not include details of how circulating cells types are involved in D^+^HUS, but rather centers on resident cells of the kidney. 

## 2. Thrombotic Microangiopathies (TMAs): The Relationship of D^+^HUS, D^-^HUS and TTP

The association of *E. coli* Shiga toxins with diarrhea-associated hemolytic uremic syndrome (D^+^HUS) was established in 1985 [1]. For years, a lack of mechanistic information complicated efforts to understand the causes of the other TMAs. Some pertinent reviews of these TMAs are listed [2,3,4,5,6,7,8,9,10,11]. Fortunately, recent developments in the basic science of the TMAs have provided a causal separation for these TMAs. Clinical symptoms of these three diseases are overlapping, and all appear to have damaged microvascular endothelium as a primary feature. D^+^HUS is caused by the action of Stx on multiple cell types in the kidney whereas D^-^HUS (atypical HUS) is caused by dysfunctional complement regulatory proteins, and TTP is initiated by deficient ADAMTS13 protease activity for degradation of platelet-activating ultra large von Willebrand factor (vWf) multimers. 

Despite the distinct initial causes of each, there are hints of biological mechanisms that may overlap in the disease processes. For example, it is not entirely clear if altered complement activity, a key feature of D^-^HUS, or abnormal von Willebrand factor in TTP also have a role in predisposing some individuals to the action of Shiga toxin in D^+^HUS (typical HUS) [12,13]. This also opens the door for the potential role of genetic predisposition for D^+^HUS. Such genetic predisposition exists for complement regulatory factor proteins in D^-^HUS and for ADAMTS13 protein, a von Willebrand factor cleaving protease in TTP [5,14,15,16,17]. It is important to note that the need remains to determine the specific cause of each of the individual hallmarks of TMA; thrombocytopenia, microangiopathic hemolytic anemia, and acute renal failure. 

Another very important component of these diseases is the neurological sequelae. The causes of the changes in CNS function are the least studied among of the TMAs. Although the endothelium remains a focal point here as it does for the corresponding renal disease, new findings in D+HUS indicate the neuron is a plausible target for Shiga toxin in the CNS [18,19,20,21].

In this review, the animal models discussed are referred to a D^+^HUS models, although some of these do not include a diarrheal phase. However, they all result in renal disease related to Shiga toxin action and exhibit aspects of D^+^HUS in humans.

## 3. Time Course Development of D+HUS

An accurate timeline for D^+^HUS was derived from a large prospective clinical patient referral study of children in the Pacific Northwest [22]. Three days after ingesting STEC-contaminated material, individuals develop moderate diarrhea and significant abdominal pain. Approximately 3 days later, bloody diarrhea develops in most of these individuals prompting medical attention. It is here that a stool sample is taken for analysis of STEC and Shiga toxin. Importantly, it is during the hemorrhagic colitis stage that Stx1 and/or Stx2 enter the blood circulation setting in action a series of toxemic reactions that culminate in renal failure in 5–15% of the patients. STEC does not colonize the blood, thus D^+^HUS is a toxemic rather than a bacteremic event. The toxemic period advances to acute renal failure in approximately 4 days after the hemorrhagic colitis phase. Fortunately, most patients resolve the systemic complications and do not progress to renal failure. Although the latter 4 days represent a potential ‘therapeutic window’, there is no therapeutic treatment other than fluid volume control and dialysis currently available to reduce or prevent renal failure in D^+^HUS. Another complicating factor in D^+^HUS is that antibiotics are not recommended in the earlier phases, *i.e.*, prior to appearance of bloody diarrhea because STEC bacteria respond to some antibiotics by producing excess Shiga toxin [23,24]. The topic of therapeutics is briefly addressed in a section below for identification of therapeutic targets to prevent renal damage. 

## 4. Shiga Toxin-Producing E. coli (STEC)

This form of *Escherichia coli* (*E. coli*) bacteria is a pathogen associated with contamination of food and water. Equivalent terms for this *E. coli* are Shiga toxin-producing *E. coli* (STEC), or Verotoxin-producing *E. coli* (VTEC). The primary virulence factor in systemic host responses produced by clinical isolates of STEC is Shiga toxin type 2 (Stx2), but some isolates produce both Stx1 and Stx2, or more rarely only Stx1 [25,26,27]. Numerous other factors produced by STEC are believed to act locally in the intestine rather than systemically as do the Shiga toxins. The reservoir for STEC is primarily bovine, however other domestic and wild animals with rumen digestive systems are all capable of harboring STEC [28,29]. It appears that Stx does not cause disease in these animals due, in part, to a paucity of functional Stx receptor in the animals [30]. STEC is an efficient pathogen in humans as ingestion of less than 100 bacteria is sufficient to colonize the colon. Thus, ground beef, water, or vegetables contaminated with bovine feces are a risk to the public as are contaminated unpasteurized milk or apple cider. The most common form of STEC is E. coli O157:H7, although there are other E. coli serotypes that produce Stx1 and/or Stx2 which cause D^+^HUS [31,32]. 

## 5. Shiga Toxin Structure and Function

It is commonly accepted that Shiga toxin (Stx) is the primary virulence factor of pathogenic STEC bacteria in D^+^HUS [1,33,34]. Evidence for the total dependence on Stx for the vascular effects of disease was provided in a lower primate model [35]. In this study, a comparison was made in host responses following oral inoculation with wild type Stx-producing pathogenic bacteria *vs.* with a Stx-deficient strain of the pathogen. The intestinal hemorrhagic response was observed only with the wild-type bacteria. The following is an abbreviated description of structure and functions of the Shiga toxins. 

### 5.1. Structure of Shiga Toxin

All of the Shiga toxins comprise a single 30 kDa A-subunit and a pentamer of non-covalently attached identical 7 kDa B-subunits [36]. Enzymatic activity resides in the A-subunit whereas the cell recognition receptor binding properties are in the B-subunits. The Stx receptor, Gb3, is described in more detail in the following section. The genes for Shiga toxin are located on a bacteriophage (a virus of bacteria) that is associated with all pathogenic STEC. The toxin gene is transcribed and its messenger RNA translated with subsequent assembly of the holotoxin for export to the periplasmic space in the bacteria or secreted by the bacteria. 

### 5.2. Stx1 vs. Stx2

Despite related primary amino acid sequences, Stx1 and Stx2 are immunologically distinct [33]. In addition, Stx1 and Stx2 do not target exactly the same tissues and organs although both bind Gb3 and are capable of causing D^+^HUS [37,38,39]. These properties are realized in animal models of disease where Stx2 is many times more potent than Stx1 in mice with the reverse being true in rabbits [40]. However, Stx2 is most commonly associated with clinical isolates of STEC and thus is believed to be the primary form of importance in human disease. 

Unfortunately, there is no universal source of highly purified toxin available to all researchers that would eliminate the variability in experiments from different laboratories. Some commercial sources of these toxins are only 50% pure, yet results from experiments performed with such toxin are occasionally reported to be due to the toxin itself. This remains a problem and is one that actually provides a valid explanation for some disparate results among related experiments. LPS is a much more potent pro-inflammatory agent than Stx and results due to LPS *vs.* Stx is valuable knowledge for an understanding of D^+^HUS. Thus, it is important that Stx1 and Stx2 preparations be processed to remove contaminant proteins and residual LPS, and prior to use the final toxin preparation should be analyzed by silver staining following gel electrophoresis, by dose-response in a Vero cell cytotoxicity assay, and by neutralization of biological activity with toxin-specific monoclonal antibodies in the Vero cell assay. Interpretation of data should also take into account the amount of Stx employed in animal experiments. In some cases, there has been a 10 to 40-fold difference in the amount of Stx2 that has been used for murine experiments. The data derived from studies using 10 or 20-times the lethal dose of toxin is likely to be less useful for interpretation purposes. 

### 5.3. Shiga Toxin Internalization Processing, Enzymatic Activity, and Cellular Responses

Following binding to Gb3 on target eukaryotic cells, the Stx-receptor complex is internalized and locates within endosomes. Rather than moving to lysosomes for degradation, the complex is transported in a retrograde manner through the golgi to the endoplasmic reticulum (ER) [41]. It is here that the Stx A-subunit is cleaved by a furin-like protease, releasing the enzymatically active A1-subunit into the cytoplasm where it exerts its effect on ribosomes [42]. Alternatively, there is evidence that internalized Stx-Gb3 can be transported directly to nuclei [43,44]. However, in the latter case, the action of Stx in the nucleus has yet to be delineated. 

The Shiga toxins enzymatically inactivate the eukaryotic ribosome by removal of a single adenine base from 28S rRNA within the large (60S) ribosomal subunit [45]. This is an irreversible process that renders the ribosome defective for interaction with eukaryotic peptide elongation factor for binding of aminoacyl-tRNA and elongation of nascent peptide [46]. 

Shiga toxin can exert its effects on eukaryotic cells by one of three known mechanisms. Firstly, inactivation of ribosomes and inhibition of cytoplasmic protein synthesis can result in cell death [47]. Secondly, Shiga toxin-dependent generation of the depurinated 28S rRNA in ribosomes initiates a unique signal-transduction response known as the ‘ribotoxic stress response’ (RSR) that leads to activation of cytokines, chemokines, or other factors that result in numerous different events including apoptosis of the affected cell [48]. The signaling pathways activated in the RSR include the mitogen-activated protein kinases (MAPK) such as p38MAPK [48,49,50,51]. Interestingly, these activities appear to be due to other than inhibition of protein synthesis. Thirdly, receptor binding of Stx holotoxin or its B-subunit alone can initiate a cytoplasmic signal-transduction cascade different from the RSR response-activated pathway [52,53]. This process requires one or more factors, likely membrane proteins with a cytoplasmic domain because Gb3 itself is incapable of initiating cytoplasmic signaling. Recently, candidates for the ‘unknown’ factor have been suggested [52,54,55,56].

Thus, Stx can exert different responses in a cell type-specific manner. The end results of these events can be cell death (apoptosis, necrosis) or inflammatory responses in cells that remain viable, and perhaps other intermediate responses.

## 6. Shiga Toxin Receptor, Globotriaosyl Ceramide (Gb3)

The Stx receptor is a major determinant and of central importance to D^+^HUS kidney disease [57,58,59,60]. The receptor for Stx1 and Stx2 is a neutral glycosphingolipid, globotriaosylceramide, abbreviated Gb3. Gb3 is synthesized in golgi of select eukaryotic cells and transported to the plasma membrane where it resides in the outer leaflet with its trisaccharide moiety facing outward and the hydrocarbon ceramide (C-16 to C-24) moiety non-covalently arranged within the plasma membrane. The binding subunit of Stx specifically recognizes the terminal alpha-1,4 di-galactose of the trisaccharide. A molecule of Stx contains five binding (B-) subunits, each capable of binding one or more molecules of Gb3, resulting in cooperative high affinity binding of Stx to cells with a Kd of approximately 0.1 nM [61,62]. The importance of Gb3 in Stx action is evident from cell culture and animal studies where the absence of Gb3 eliminates the response to Stx [63,64].

There is a relationship between the amount of Gb3 in cells and their sensitivity to Stx, although this appears not to be stoichiometric. Other receptor-related determinants of Stx sensitivity of a cell are complex biochemical aspects of the Gb3 membrane microenvironment. In brief, much or perhaps most of the Gb3 in a plasma membrane may not be reactive with Stx. This cryptic Gb3 may be a function of direct interaction of the Gb3 ceramide moiety with other membrane components including cholesterol, other glycolipids, fatty acids, or proteins [65,66,67,68]. This concept helps explain why a tissue often appears to bind much less Stx compared to the total amount of Gb3 that is extractable from the tissue. The story of Gb3 and plasma membrane microdomains is only now coming into focus [67,69,70,71,72]. Thus, Gb3 molecules with identical trisaccharides, but different ceramides, may be arranged in membranes differently leading to their differential interaction with Stx1 *vs.* Stx2. It is more difficult to accept that two molecules of Gb3, identical in all respects, may have differential preference for the toxins. However, as described above, different domains of plasma membranes being unique appear to influence the presentation of identical Gb3 molecules and reactivity with Stx1 or Stx2.

What this means for D^+^HUS kidney disease is that it remains important not only to determine where and how much Gb3 is localized to different renal cell types, but also if the Gb3 is reactive with Stx1 or Stx2. Finally, the host response to Stx should represent a change in physiology consistent with the known function of the cell type. 

## 7. Shiga Toxin Receptor Localization in the Kidney

Differential expression of Stx receptor (Gb3) by cell types of human and animal kidney is a reliable predictor of Stx site of action. Gb3 has been detected in homogenates of total kidney from human and animals. In these cases, detection methodologies include thin-layer chromatography with detection of Gb3 by Stx overlay, high-performance liquid chromatography, or mass spectrometry of extracted total neutral glycolipids [73,74]. In addition, Gb3 has been localized within kidney tissue samples by anti-Gb3 antibody reactivity or by detection of bound Stx using anti-Stx antibodies [73,75,76].

### 7.1. Stx Receptor Localization in Glomeruli

Human glomerular cell types that express Stx receptor include: endothelial, podocyte, and mesangial. It is likely that these cell types in baboon glomeruli also express the Stx receptor. Porcine glomerular endothelial cells are positive for Gb3 [77,78,79]. Glomeruli from Dutch belted rabbits, but not from New Zealand White rabbits appear to develop lesions following oral infection with STEC [80,81,82,83]. Murine, and perhaps all rodent glomerular cells do not express Stx receptor [12,39,84]. 

### 7.2. Stx Receptor Localization in Extra-Glomerular Regions

In humans, and most animal models of D^+^HUS, Gb3 is expressed by proximal tubules [39,85,86,87]. Porcine and murine renal tubules express Stx receptor and bind Stxs [78,79]. Human and murine collecting duct cells also express Gb3 [84]. A systematic study of *in situ* Gb3 localization in other epithelial cells of the nephron in human or animal kidneys has not been completed as this requires demonstration of co-localization of Gb3 with markers specific for each cell type along the nephron. It has been reported that in murine kidneys, Gb3 co-localizes with aquaporin-2, a marker of collecting duct cells [84]. Unexpectedly, murine collecting ducts express more Gb3 than do proximal tubules. 

## 8. Animal Models of D+HUS

### 8.1. General Comments

It is not the purpose of this review to provide details of animal models of D+HUS [88]. Animal models of D^+^HUS have been employed for the salient reason that human studies are limited to analysis of blood and urine samples, a rare renal biopsy or autopsy sample. Prior to animal models, the discussions of mechanisms of D^+^HUS were based largely on the analysis of human renal tissue autopsy samples. However, autopsy samples represent late stage changes which are inherently less helpful for determining the mechanisms of pathogenesis. The ideal animal model of D^+^HUS would start with oral inoculation of fully virulent STEC and end with complete D^+^HUS. For a number of reasons, this ideal model does not exist. Nonetheless, much valuable information has been obtained from animal models of D^+^HUS that begin with either oral inoculation of the pathogen or with direct intraperitoneal or intravascular injection of the purified toxin. 

The most common animals utilized in D^+^HUS studies are the mouse, rabbit, pig, and baboon. The following are some generalities generated from the D+HUS animal studies of many different groups. Firstly, each animal model is distinct and different in response to either STEC bacteria or purified Stx1 or Stx2. Secondly, the animal models differ in location of the organ damage (*i.e.*, kidney, intestine, CNS) which most closely represents that of human D^+^HUS. Thirdly, the mouse has been the most utilized D^+^HUS model for baseline knowledge, cost, availability of reagents, and genetic applications [40,89,90,91,92,93,94,95]. Fourthly, the baboon D^+^HUS model most closely represents D^+^HUS of humans and for this reason will continue to be important for validation of data derived from the small animal models of D^+^HUS [96,97,98,99,100]. 

### 8.2. Mouse

Results from the mouse model of D^+^HUS have been instructive for kidney disease whether beginning from oral STEC or injected (i.p. or i.v.) Stx1 and Stx2 [39,76,90,92,93,95,101,102,103,104]. A mouse-specific strain of STEC exists, but has yet to be studied in detail in mice for renal disease, although the pathogen-free mouse inoculated with STEC has yielded information of renal disease [93,94]. The mouse D^+^HUS model also has been useful for application of host gene-arrays in analysis of renal responses, something that is not yet available for rabbit, pigs, or baboons [92]. The primary disadvantage of the mouse model of D^+^HUS is that mice, and perhaps all rodents, do not express Stx receptor on their endothelium [12,84]. It appears that some vascular changes in the murine D+HUS model require the combined action of Stx2 and LPS [92,105,106]. The renal coagulation and thrombosis observed in the Stx2/LPS mouse model is driven by LPS induction of chemokines in proximal tubule cells with Stx2 prolonging the half-life of the chemokine mRNAs [92,105]. Neutralization of the chemokines with monoclonal antibodies inhibited this coagulation and thrombosis. 

### 8.3. Rabbit

The Dutch Belted rabbit develops acute renal failure following exposure to STEC, but exhibits a low and variable rate of response [80,81,82]. A naturally existing rabbit-specific variant of STEC discovered in Dutch-belted rabbits remains a source of inoculum for renal disease studies and is adaptable to other types of rabbits [80]. 

### 8.4. Pig

The pig model of Stx-induced renal disease has not been described in detail, but it demonstrates tubular damage [79,107,108,109]. A natural STEC that produces Stx2e is associated with systemic disease in piglets [110]. 

### 8.5. Baboon

Finally, the baboon appears to be the ‘acid test’ model of D^+^HUS for kidney failure because the physiology most closely represents that of the human kidney [96,97,98,99,100,111]. The primary disadvantages of the baboon for this purpose are its difficult use for oral inoculation studies and lack of species-specific gene microarrays. Results of STEC or Stx related kidney damage in these animal models are discussed in more detail below. 

## 9. The Kidney as a Target of Shiga Toxin

### 9.1. Why the Kidney?

It is not totally clear why the kidney, aside from the CNS, is the most affected organ in D^+^HUS. Although Stx from the intestine would traverse the lung prior to encountering the kidneys, damage to the lungs in D^+^HUS is secondary in severity to that of the kidneys. A plausible explanation is the kidney expresses the most abundant amount of biologically active Stx receptor and thus contains the most Stx-sensitive cell types. Another reason is the high volume of blood flow and filtration rate of blood in kidneys increase the chance of Stx interaction with cells of the renal microvasculature and the filtration barrier. Indeed, as described in more detail below, human renal endothelial and podocyte cells are very sensitive to the action of Stx2 [84]. In addition, should the renal filtration barrier become damaged, Stx would have an opportunity to interact with the different tubular epithelial cells types which comprise the nephron. Again, there is evidence that some of these latter cell types do express sufficient Stx receptor to be candidates for Stx-induced disease.

### 9.2. Differential Sites of Action for Stx1 vs. Stx2.

Injection of animals with Stx1 or Stx2 results in preferential damage to organs including kidney and lung. Stx1 appears to target the lung while Stx2 prefers the kidney [39]. A data-based explanation for this phenomenon is beginning to come forward as more is revealed about the biological relationship of Gb3 with plasma membranes [65,66,67,71,112,113]. A complete study of the differential effects of Stx1 *vs.* Stx2 on kidney has not been performed in animal models of D+HUS. 

## 10. Renal Cell Types Sensitive to Stx

### 10.1. General Comments

An ongoing process in studies of D^+^HUS is to digest the large volume of data derived from human renal cells *in vitro*, and to connect these data in a meaningful way with information about renal damage in humans. A recent review describes the use of different renal cell types in the study of renal disease [114]. There is good reason to believe that certain human renal cell types would be an important part of the D^+^HUS disease process should they express Gb3, be sensitive to Stx *in vitro*, and are located within a part of the kidney identified with the pathological disease in D^+^HUS. To date, these include human podocyte, endothelial, mesangial, proximal tubule and collecting duct tubule cells. These are discussed in more detail below. 

### 10.2. Renal Endothelial Cells

If there is any consensus among clinicians and researchers in the field of D^+^HUS, it is that the endothelium is a likely target of Stx [115]. Examination of renal pathological samples typically reveals swollen and detached glomerular endothelium. The complementary data supporting the concept that endothelial cells represent a primary target in D^+^HUS was provided in a Stx1 cytotoxicity assay with human umbilical vein endothelial cells, HUVEC [47,116]. In these studies, a series of primary cell isolates were prepared from individual umbilical cords. Although most cell isolates were relatively resistant to Stx (LD50 = 1nM Stx), it was noted that approximately 7% of the isolates were 10 to 100-times more sensitive to concentrations of Stx1 [117]. This was a stable phenotypic property of the HUVECs. Although large vessel endothelium is not damaged in D^+^HUS, this observation could possibly be related in terms of genetic predisposition to the similar percentage of individuals who progress to renal failure in D^+^HUS. Subsequent studies showed that most HUVEC express little Gb3 compared to human microvascular renal endothelial cells, thus providing an explanation of the generally poor sensitivity of large vessel *vs.* microvascular endothelial cells [118,119]. A conceptual disconnect remains in that human renal microvascular cells (HRMEC) are exquisitely sensitive to the lethal effects of Stx *in vitro*, but *in vivo* HRMEC only become swollen and detached from the basement matrix in D^+^HUS. This suggests the presence of a mechanism that partially protects renal endothelium from Stx *in vivo* that does not exist *in vitro*. The biological cross-talk between the glomerular fenestrated endothelium and podocytes may be important to this phenomenon [120]. As with other different diseases of the kidney, damage to individual glomeruli is less than uniform or complete in D^+^HUS.

Animal models of D^+^HUS differ in their response of glomerular endothelium to the direct action of Stx. Importantly, baboon, rabbit and pig express Stx receptor on their microvascular endothelial cells while mice do not [77,84,96,98,121]. Thus, in the mouse models of D^+^HUS, all of the changes in biology and pathology of renal endothelium related to Stx must be indirect. Some insight into how this might occur is provided below, particularly for the observed coagulation and thrombosis [92,93,105]. Given these differences for the renal endothelium among animals, it is not surprising that some animal models of D^+^HUS are associated with more complete occlusion of glomerular microcapillaries [12,83,92,93,96,99,122,123,124,125,126].

The glomerular endothelium, being part of the filtration barrier of the kidney, when damaged can become leaky and may, in part, be responsible for proteinuria and appearance in the urine of host cytokines, chemokines, and other biological markers of disease. However, some inflammatory mediators appear to be of renal origin in D+HUS. 

### 10.3. Podocytes

Human podocytes (visceral epithelial cells) are almost as sensitive to Stx2, *in vitro*, as are human renal microvascular endothelial cells and they also express an ample amount of Stx receptor [84]. Analysis of human D^+^HUS pediatric kidney on autopsy revealed Stx binding to podocytes [127]. As with endothelial cells, mouse podocytes also do not express the Stx receptor and are insensitive to Stx2 [84]. However, the Stx-sensitivity status of podocytes in other animals is not as clear. The primary role of podocytes includes participation in the blood filtration barrier along with glomerular endothelium and basement membrane [128,129]. Podocytes also provide VEGF in support of glomerular endothelium [120,130,131,132] and utilize VEGF beneficially in an autocrine manner [133].

Alteration of podocytes is a feature of several well-known renal diseases including focal segmental glomerular sclerosis (FSGS), polycystic kidney disease (PKD), diabetic nephropathy (DN) [134,135,136]. Damaged podocytes have also been suggested as a key component of preeclampsia which shares some clinical features with D^+^HUS [137,138]. Stx was reported to increase endothelin-1 in human podocytes [139]. Additional studies are needed to determine the relative importance of podocytes as a target of Stx in D^+^HUS. However, biomarkers of podocyte damage have been detected in urine of D^+^HUS patients [140,141]. Importantly, Stx2 decreases VEGF production by human podocytes, *in vitro* [84]. 

### 10.4. Mesangial Cells

It is often reported that the pathology of human D^+^HUS kidney includes mesangial expansion or proliferation [142,143,144]. These glomerular cells bind and internalize Stx *in vitro*, with reported changes in mesangial physiology, but the relative role of mesangium in Stx-induced glomerular disease is less studied than for other renal cell types [127,143,145,146,147]. Animal D^+^HUS model studies have reported changes in the rabbit mesangium, but not in the murine or baboon models [81,92,93,96]. 

### 10.5. Proximal Tubular Cells (PTC)

Many reports place proximal tubular cells at the top of the list along with endothelial cells for renal cell types that respond to Stx and are of importance in D^+^HUS. Due to the availability of primary and immortalized cell lines, human PTCs are the most studied in D^+^HUS. Gb3 has been localized to PTCs from human and animal kidneys [39,86,148]. Responses of PTCs to Stx1 and Stx2 include inhibition of protein synthesis, induction of cytokines [86,148], chemokines [105], and tissue factor [87], apoptosis [149], necrosis [85,86,95,101,150], and inhibition of water reabsorption [151]. The normal function of proximal tubule cells is to reabsorb urinary solvents and solutes which pass through the glomerular filtration barrier so as to maintain proper volume and solute concentrations in the blood circulation [152]. A complicating factor is that inflammation and related signaling pathways become activated in podocytes exposed to excess protein [153,154,155].

### 10.6. Collecting Duct Cells

Collecting duct cells in human kidneys were reported to express Gb3 [75]. In murine kidney, these cells express ample Gb3 that co-localizes in the plasma membrane with aquaporin 2, a specific marker of collecting duct cells, and respond to Stx2 in an apoptotic manner [84]. This also has implications for the renal features of D^+^HUS as the collecting duct cell functions to maintain water balance of the body. In D^+^HUS patients (and most animal models), dehydration is a prominent feature, presenting as oliguria and anuria which must be treated. Initial data suggest that prevention of Stx2-dependent damage to these cells helps preserve renal function in the murine model of D^+^HUS [84]. 

## 11. Why is the Incidence of D+HUS More Common in Children?

Most D^+^HUS is associated with young children although individuals of all ages can develop D^+^HUS. A simple explanation would be that more Gb3 is expressed in the kidneys of the young. However, this has proven not to be the case as there is more Gb3 extractable from kidneys of adults *vs.* children [60,75]. An alternative explanation is that pediatric kidney expresses more Gb3 on cells that are more involved in biological responses leading to D^+^HUS. It is interesting that only pediatric kidney expressed Gb3 in glomeruli [75]. An independent verification of these data would be helpful. Information is only now becoming available in support of another concept, that the plasma membrane microenvironment nearby Gb3 dictates the biological response to Stx [65,66,67,68,70,156,157]. Such conjecture can be carried further to include other variables such as differential trafficking of the Stx-Gb3 complex inside target cells and/or activation of signal transduction pathways that are more damaging to the target cell. Although reports on these sub-topics are needed, methodologies are currently available to sufficiently provide answers to these problems. Others have reported that an ‘immunological memory response’ may be responsible for the higher incidence of D^+^HUS in children [158,159]. It is reasonable that more than one of these mechanisms may be functional in pediatric D^+^HUS.

## 12. Is There a Role for E. coli Lipopolysaccharide (LPS) in D+HUS?

Humans with D^+^HUS develop circulating antibodies to STEC LPS [160,161,162]. Thus, the blood may serve as a delivery system not only for Stx, but for LPS as well. Importantly, LPS is a much more potent pro-inflammatory agent than either Stx1 or Stx2. For example, LPS caused significant changes in the activities of many more genes of the murine kidney than did Stx2 [84]. Due to the ubiquitous distribution of LPS receptor (TLR4) in most host tissues and in cells along the nephron, there is ample reason to believe that LPS from STEC may be involved in some aspects of D^+^HUS [163]. This becomes more apparent considering documented direct interaction of LPS with the kidney [164,165,166,167,168]. However, quantitative data regarding LPS in the blood of D^+^HUS patients is needed. In the murine model of D+HUS, one of the three hallmarks of D^+^HUS was driven by LPS rather than be Stx2 [92]. In addition, some human renal cell types respond directly to LPS [163,169,170,171,172,173,174,175,176]. It is clear that activation of platelets in D^+^HUS requires initial interaction with LPS [177,178,179]. Murine and human glomerular endothelial and podocyte cells respond to LPS [84]. Some actions of LPS *in vitro* are not restricted to LPS from STEC, but appear to require the lipid A moiety (endotoxin) common to all E. coli LPS [180]. There is evidence that LPS enhances the actions of Stx in animal models of D^+^HUS [89,106,174,177,179,180,181,182,183,184,185,186,187,188]. LPS has been associated with induction of Gb3 in human endothelial cells *in vitro* [180], and in baboon renal glomerular endothelial cells, *in vivo* [111]. In the murine model of D^+^HUS, a study of temporal renal responses demonstrated early (0–24 h) changes were due to LPS and later (24–96 h) changes were due to Stx2 [92]. In addition, these gene microarray data showed the renal responses to LPS and Stx2 appeared to be additive rather than synergistic. More studies are needed to further define the specific responses to LPS *vs.* Stx2 throughout the kidney and to determine the level of circulating LPS in D^+^HUS patients. 

## 13. The Role of Circulating Cell Types in Renal Disease of D+HUS

### General Comments

Several cell types in the blood circulation have been implicated in different aspects of D^+^HUS. The more prominent aspects of this sub-topic are the following. Although controversial, circulating neutrophils have been implicated in binding and trafficking of Stx in humans and animals [189,190,191,192,193]. Data exist supporting or refuting an essential role of neutrophils and/or monocytes in D^+^HUS. Monocytes and macrophages may be a source of cytokines and chemokines in D+HUS [105,194,195,196,197,198]. Finally, platelets certainly play a central role in D^+^HUS not only because thrombocytopenia is one of the hallmarks of the disease, but for their active role as inflammatory mediators [177,178,179]. Conversely, some chemokines elicited by Stx in endothelial or monocytic cells can serve as co-activators of platelets [106,184,199]. 

## 14. Conclusions

Renal aspects of D^+^HUS are complex due to the numerous variables in primary, secondary, *etc.*, targets of Stx and the timing of responses to both Stx and LPS. We now know considerably more about the biochemical mode of action of Stx than we do about the disease process it causes. However, much has been learned about the role of Shiga toxin and LPS in the renal physiology and disease in D^+^HUS. Most of the renal cell types involved in the disease have been identified and their respective contributions are being revealed. Strides have been made in equating the animal model data with D+HUS of humans. An active movement is on to determine the relative importance in D+HUS of factors known to function in D^-^HUS and TTP, such as complement regulatory proteins and vWf proteases. Finally, the field has advanced sufficiently so as to identify therapeutic targets in D^+^HUS with the hope of impeding or preventing renal failure itself.
